# Identification of Laccase Family of *Auricularia auricula-judae* and Structural Prediction Using Alphafold

**DOI:** 10.3390/ijms252111784

**Published:** 2024-11-02

**Authors:** Jeong-Heon Kim, Youn-Jin Park, Myoung-Jun Jang

**Affiliations:** 1 Department of Plant Resources, Kongju National University, Yesan 32439, Republic of Korea; zxcb213@smail.kongju.ac.kr; 2 Legumes Green Manure Resource Center, Kongju National University, Yesan 32439, Republic of Korea; parkyj@kongju.ac.kr

**Keywords:** *Auricularia auricula-judae*, de novo sequencing, laccase, protein secondary structure, protein tertiary structure

## Abstract

Laccase is an enzyme that plays an important role in fungi, including lignin degradation, stress defense, and formation of fruiting bodies. *Auricularia auricula-judae* is a white-rot fungus in the Basidiomycota phylum, capable of delignifying wood. In this study, seven genes belonging to the laccase family were identified through de novo sequencing, containing Cu-Oxidase, Cu-Oxidase_2, and Cu-Oxidase_3 domains. Subsequently, the physical characteristics, phylogenetic relationships, protein secondary structure, and tertiary structure of the laccase family (*AaLac1*–*AaLac7*) were analyzed. Prediction of N-glycosylation sites identified 2 to 10 sites in the laccase family, with *AaLac7* having the highest number of sites at 10. Sequence alignment and analysis of the laccase family showed high consistency in signature sequences. Phylogenetic analysis confirmed the relationship among laccases within the family, with *AaLac3*–*AaLac4* and *AaLac5*–*AaLac6* being closely positioned on the tree, exhibiting high similarity in tertiary structure predictions. This study identified and analyzed laccase family genes in *Auricularia auricula-judae* using de novo sequencing, offering a simple method for identifying and analyzing the laccase family in organisms with unknown genetic information.

## 1. Introduction

Laccase, a type of Blue multicopper oxidase (MCO) (p-diphenol:dioxygen oxidoreductase, EC 1.10.3.2), utilizes the oxidizing power of copper ions to reduce molecular oxygen to water and catalyze the oxidation of various aromatic substrates [1]. Laccase contains one type-1 (T1) copper (Cu1) and one type-2 (T2) copper (Cu2), as well as two type-3 (T3) coppers (Cu3), with Cu2 and Cu3 forming a trinuclear cluster [2]. The primary role of the T1 copper is to shuttle electrons from the substrate to the cluster, while the trinuclear cluster performs oxygen reduction [3,4].

Laccase is widely distributed in plants, insects, fungi, and bacteria and is encoded by a multi-gene family [5]. For example, a model plant *Arabidopsis thaliana* has 17 genes encoding laccase, while *Nephotettix cincticeps* has 5 genes, and *Trametes villosa* has 3 genes [6,7,8]. In plants, laccase is involved in lignin polymerization [9,10], whereas in fungi, it plays crucial roles in lignin degradation, morphogenesis, stress defense, plant-pathogen/host interaction, and sporulation [1,11]. Most lignin-degrading fungi produce at least one laccase isoenzyme, and laccase is the primary enzyme influencing lignin degradation in soil environments [12]. Particularly, laccase exhibits effective capabilities in lignin degradation as it does not require H_2_O_2_ in the lignin degradation process [13].

Laccase is involved in the formation of fruiting bodies in basidiomycetes and ascomycetes [14,15,16]. According to reports, *Schizophyllum commune*, *Volvariella volvacea*, and *Agaricus bisporus* have shown associations with laccase in fruiting body formation [17,18,19]. Some fungal laccases are involved in the synthesis of 1,8-dihydroxynaphthalene (DHN)-melanin, and several byproducts of the DHN-melanin biosynthesis pathway possess antifungal or immunosuppressive properties [20,21].

*Auricularia auricula-judae*, belonging to the Auriculariales, is a mushroom widely consumed worldwide due to its excellent taste and nutritional value [22,23]. *A. auricula-judae*, as a species of white-rot fungus, possesses the ability to delignify plant residues [24]. Previous studies have reported its capability to decompose forestry residues, suggesting its potential in the production of food, fermentable sugars, and solid fuels [25].

The tertiary structure of proteins plays an essential role in determining function by forming active sites and facilitating substrate binding [26]. For this reason, substantial research has been conducted to predict tertiary structures based on protein amino acid sequences [27]. AlphaFold, a recently developed AI (Artificial Intelligence)-based program, predicts protein structures with high accuracy from amino acid sequences [28]. The tertiary structures predicted by AlphaFold can be used to predict protein–protein interactions, protein–ligand complexes, and protein–nucleic acid complexes, providing valuable information for biotechnology industries, including pharmaceuticals and healthcare [29,30,31]. Meanwhile, *A. auricula-judae* has not undergone whole-genome sequencing, and its genome annotation is not available. Thus, we obtained unigenes through de novo sequencing and identified the presence of Cu-Oxidase domains using a Hidden Markov Model (HMM) to detect members of the laccase family.

This study identified the laccase family of *A. auricula-judae* through RNA de novo sequencing. Subsequently, we classified each laccase based on sequence and structural features and predicted the tertiary structure of the laccase family using AlphaFold.

## 2. Results

### 2.1. A. auricula-judae Laccase Gene Family

The laccase gene family in *A. auricula-judae* consists of seven genes identified from the RNA sequencing, named *AaLac1* through *AaLac7*. Essential information for the 7-laccase family is provided in Table 1. Using NetNGlyc-1.0 to predict the number of variable N-glycosylation sites, *AaLac1* had 2 sites, while *AaLac7* had 10 sites. Information regarding the secondary structure analysis of proteins is provided in Table 2. Analysis of the secondary structure of *AaLac* revealed that they are composed of 3.52–8.16% α-helix, 25.94–30.77% extended strand, 4.9–6.01% β-turn, and 58.72–61.47% random coils.

### 2.2. Multiple Sequence Alignments and Phylogenetic Analysis

**Figure 1:**
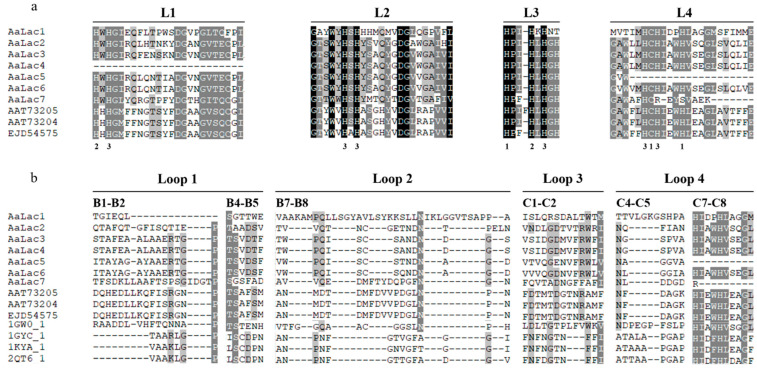
Multiple sequence alignment and structural domain analysis of *A. auricula-judea* laccases *AaLac1* to *AaLac7*: (**a**) The laccase signature sequences L1–L4. The histidine and cysteine residues are indicated according to the type of copper, with 1, 2, and 3 above representing type-1, type-2, and type-3, respectively. The amino acid sequences of *A. polytricha* laccases and *A. subglabra* laccase downloaded from NCBI are named by the GeneBank accession number. The shade levels represent conservation degrees. (**b**) To confirm the sequence of the potential substrate binding loop of the *A. auricula-judae* laccases enzyme, it was identified based on structural alignment with loops 1–4 of *Melanocarpus albomyces* (PDB code 1GW0), *Trametes versicolor* laccase I (PDB code 1GYC), *T. versicolor* laccase III (PDB code 1KYA), *Lentinus tigrinus* (PDB code 2QT6).

The amino acid sequences of laccases from *A. auricula-judae* were aligned to identify the signature sequences and substrate binding loop (SBL) (Figure 1). Analysis of predicted laccase amino acid sequences revealed that all except *AaLac4* and *AaLac5* included complete signature sequences L1–L4. In *AaLac4*, two histidines (H) were missing, while in *AaLac5*, three histidines and one cysteine (C) were deleted. Additionally, in *AaLac7*, two histidines in the L4 region were substituted with different amino acids. To predict the SBL (loop 1–4) of *A. auricula-judae* laccase sequences, they were compared to laccase sequences from *Melanocarpus albomyces*, *Trametes versicolor*, and *Lentinus tigrinus*. The SBL of *A. auricula-judae* laccases exhibit both conserved and variable regions across different laccase sequences. The SBL of *A. auricula-judae* laccases exhibit both conserved and variable regions across different laccase sequences. In the B1–B2 region of Loop 1, *AaLac3* and *AaLac4* share the same sequence, as do *AaLac5* and *AaLac6*. However, in the B4–B5 region, the sequences of *AaLac1* and *AaLac7* differ from those of other laccases in the family.

**Figure 2:**
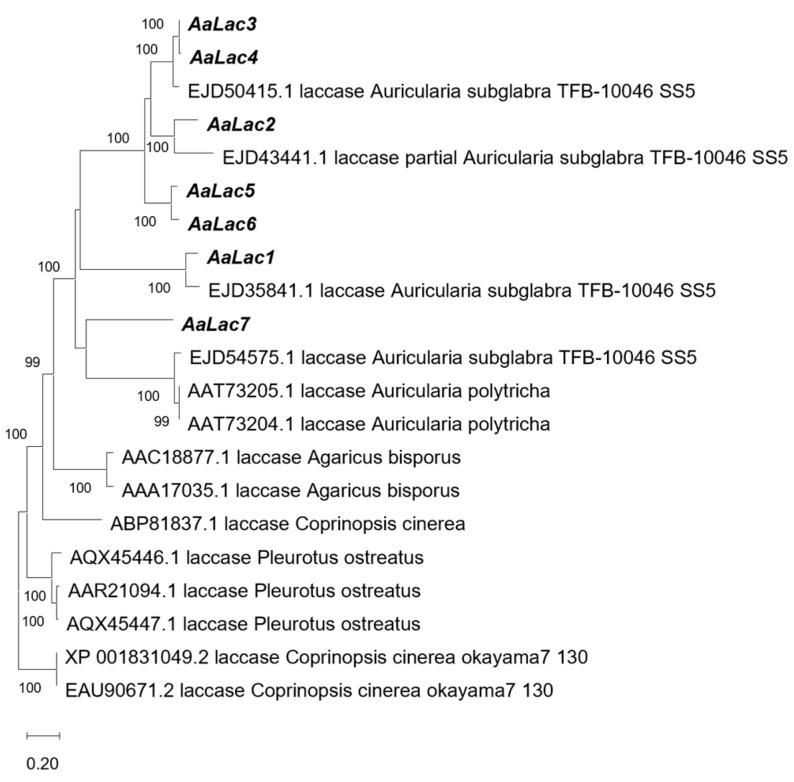
Phylogenetic tree of laccase amino acid sequences from *A. auricula-judae* and related species. Phylogenetic analysis was performed using the Neighbor-Joining (NJ) method in MEGA 11, with the Poisson model for amino acid substitution. Laccase sequences from *Auricularia subglabra*, *Auricularia polytricha*, *Agaricus bisporus*, *Coprinopsis cinerea*, and *Pleurotus ostreatus* were retrieved from the NCBI database. The laccase family identified in *Auricularia auricula-judae* is indicated in bold. Bootstrap values are based on 1000 replicates and indicate the percentage of replicate trees in which the associated laccase family clustered together. The scale bar represents 0.20 amino acid substitutions per site. Branch lengths represent the number of substitutions per site.

To investigate the evolutionary relationship between laccases in *Auricularia auricula-judae* and related species, a phylogenetic tree was constructed using the Neighbor-Joining method with the Poisson model for amino acid substitution (Figure 2). Phylogenetic analysis revealed that *AaLac3* and *AaLac4* clustered closely together, as did *AaLac5* and *AaLac6*, indicating that these pairs of laccases are closely related. In contrast, *AaLac1* and *AaLac7* appeared more distantly related to the other laccase members. Furthermore, laccase sequences from other species, including *Auricularia subglabra*, *Auricularia polytricha*, *Agaricus bisporus*, *Coprinopsis cinerea*, and *Pleurotus ostreatus*, were compared, and the laccases from the genus *Auricularia* showed high homology.

### 2.3. The Tertiary Structure of Laccase Proteins

**Figure 3:**
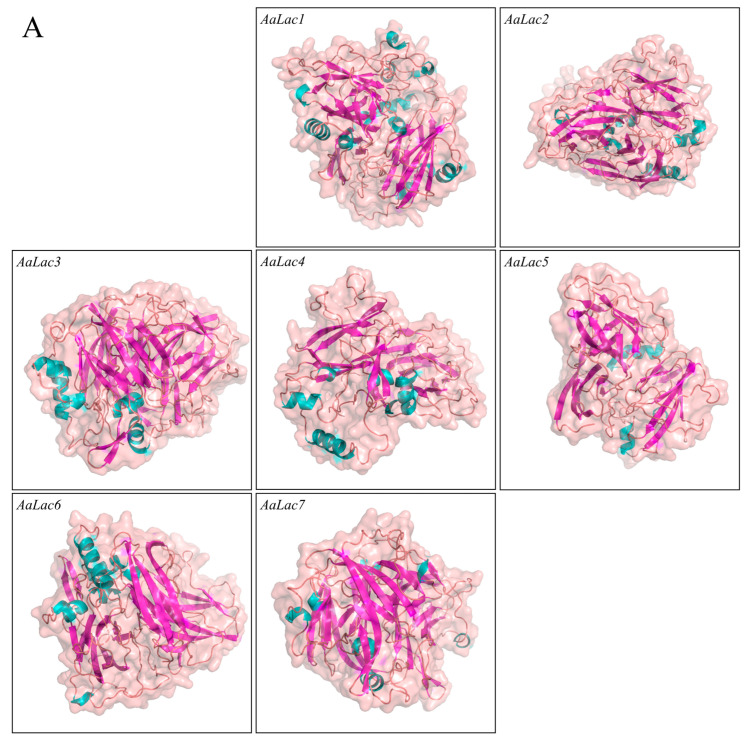

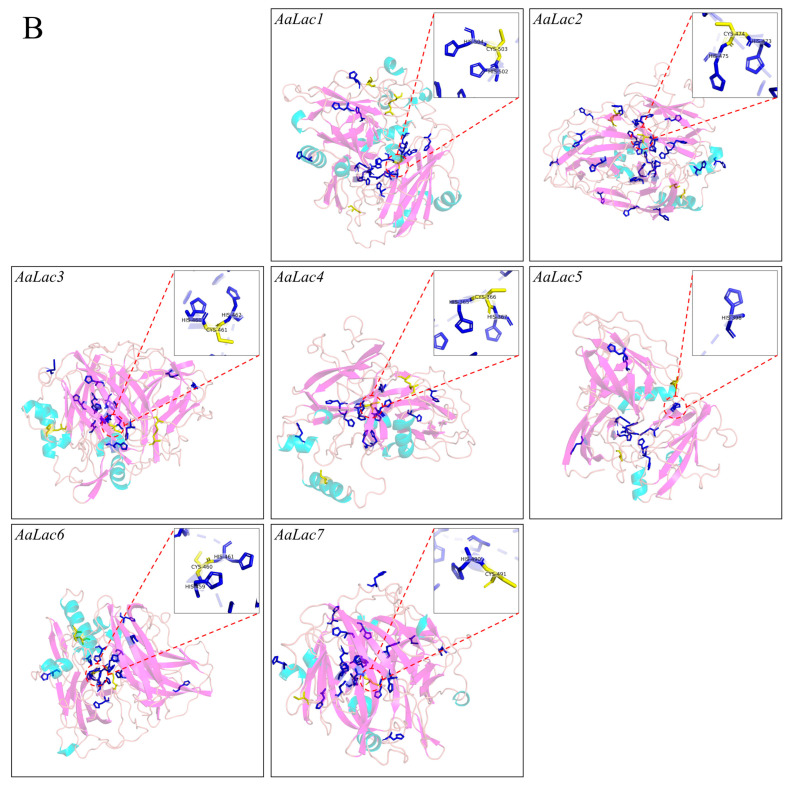
Protein tertiary structure of *A. auricula-judae* laccase family: (**A**) Proteins are in cartoon form and represented in different colors depending on their secondary structure. Helix; cyan, Sheet; magenta, Loop; tint, The protein surface is represented at 60% transparency. (**B**) Identification of histidine and cysteine residues and His-Cys-His motif in the protein tertiary structure of *A. auricula-judae* laccase family.

**Figure 4:**
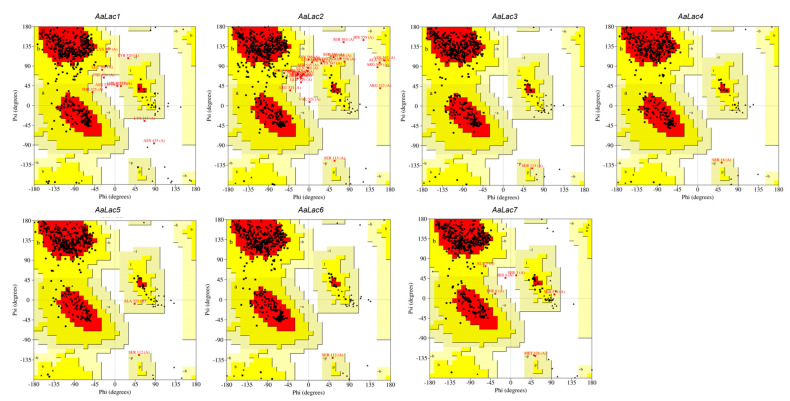
Validation of laccase family protein tertiary structures using a Ramachandran plot. Red region; most favored, Yellow region; additional allowed, Beige region; generously allowed, A; core alpha, a; allowd alpha, ~a; generous alpha, B; core beta, b; allowed beta, ~b; generous beta, L; core left-handed alpha, l; allowed left-handed alpha, ~l; generous left handed alpha, p; allowed proline, ~p; generous proline, Square; glycine, Triangle; proline.

Based on the amino acid sequences of the 7 *AaLac* proteins, the tertiary structure of the laccase protein was modeled (Figure 3A). As a result, it was observed that beta sheets predominated in the tertiary structure of all laccases, which is consistent with Table 1. The Ramachandran plot is shown in Figure 4. Ramachandran plot analysis of the laccase family showed 80.3–90.2% of amino acid residues within the most favored regions (Table 3). Additionally, ProSA Z-scores ranged from −8.55 to −6.71, and QMEAN scores ranged from 0.56 to 0.77. These protein validation metrics emphasize that the predicted models are both reliable and structurally stable. The positions of histidine and cysteine residues were identified in the tertiary structure of the laccase family, confirming the presence of the Histidine-Cysteine-Histidine (His-Cys-His) motif (Figure 3B). In *AaLac5*, where a deletion occurred in the signature sequence L4, an intact His-Cys-His motif could not be observed. Similarly, in *AaLac7*, where a substitution occurred, one histidine residue was also absent. The RMSD values among the *laccase* family ranged from 0.182 to 4.222 Å (Table 4). Specifically, *AaLac4* and *AaLac1* showed an RMSD of 4.222 Å, indicating low similarity, while all other structures showed high similarity within the family, with RMSD values under 3 Å. Notably, the RMSD between *AaLac3* and *AaLac4* was 0.182 Å, indicating high similarity, as was the case for *AaLac5* and *AaLac6*, with an RMSD of 0.228 Å. This result aligns with the trends observed in SBL and phylogenetic analysis.

## 3. Discussion

As genome sequencing technology advances, the use of mushroom genome data in research has become increasingly prevalent [32]. Whole-genome sequencing has been utilized to identify and analyze the laccase family in mushrooms such as *Agaricus bisporus*, *Flammulina velutipes*, *Pleurotus ostreatus*, and *P. eryngii* [33,34,35]. However, whole-genome sequencing generates vast amounts of data, which poses challenges for analysis [36]. In contrast, de novo sequencing targeting RNA offers the advantage of generating relatively smaller data sizes, facilitating easier and faster analysis [37]. Therefore, despite its relatively lower accuracy, de novo sequencing is being used to analyze various under-researched beneficial organisms [38,39]. To address the analytical limitations of these data, further experimental validation through PCR and Sanger sequencing will be necessary. Genes identified through sequencing can be utilized to analyze enzyme function, and recently, studies have been conducted to predict their tertiary structures and verify binding affinity with substrates through protein-ligand docking [32]. In-depth studies on the structure–function relationship of enzymes have been conducted by comparing the substrate binding affinity of normal enzymes with that of enzymes whose structure has been rearranged through mutations [33,34]. For example, artificial metalloenzymes (ArM) have shown improved protein catalytic efficiency using random mutation and targeted mutation approaches [35]. Genes identified through sequencing can be utilized to analyze enzyme function, and recently, studies have been conducted to predict their tertiary structures and verify binding affinity with substrates through protein–ligand docking [40]. In-depth studies on the structure–function relationship of enzymes are being conducted by comparing the substrate binding affinity of normal enzymes with that of enzymes whose structure has been rearranged through mutations [41,42]. For example, artificial metalloenzymes (ArM) have shown improved protein catalytic efficiency using random mutation and targeted mutation approaches [43].

Fungal laccase is involved in numerous biological processes such as morphogenesis, fungal plant-pathogen/host interactions, and stress defense, leading to extensive research focused on analyzing its functions [12,13,14,15,16]. According to KEGG pathway analysis, laccase is involved in lignin degradation, phenolic compound oxidation, detoxification, and redox cycling [44,45]. These biological activities can be analyzed through molecular docking or molecular dynamic simulation approaches. While targeted mutation and computer-assisted virtual mutants have been the primary methods used to study laccase, there is a lack of research on randomly mutated laccases and protein–ligand docking analysis. In this context, de novo sequencing can be effectively applied to discover new genetic information and analyze both structure and function.

RNA de novo sequencing of *A. auricula-judae* fruiting bodies identified seven laccase family genes containing Cu-Oxidase, Cu-Oxidase_2, and Cu-Oxidase_3 domains among the generated unigenes. N-glycosylation is the most common modification of secretory proteins in eukaryotic cells, where glycans stabilize the folded structure of proteins by promoting their folding [46]. N-glycosylation in the immune-regulating protein (FIP-glu) of Ganoderma lucidum has been shown to enhance anti-inflammatory activity by inhibiting phosphorylation of p38 MAPK [47]. Additionally, N-glycosylation in laccases has been found to affect substrate binding affinity, catalytic rates, and thermal stability [48]. Therefore, the prediction of N-glycosylation sites plays a crucial role in understanding protein function. *AaLac* proteins were predicted to have 2–10 N-glycosylation sites, with *AaLac7* having the highest number at 10. *AaLac7* can possess higher substrate-binding affinity and thermal stability compared to other laccases within the family, suggesting that further in-depth functional analysis through glycosylation inhibition experiments is needed.

Different laccase sequences within the laccase family contain four conserved signature sequences, including ten histidines and one cysteine at the copper-binding center [49]. *AaLac* also demonstrated high consistency in the signature sequences. However, some parts of the signature sequences of *AaLac4*, *AaLac5*, and *AaLac7* were not identified. In *AaLac4*, a deletion of two histidines was observed, while in *AaLac5*, deletions of three histidines and one cysteine were identified. Another noteworthy observation is that in the signature sequence L4 of *AaLac7*, two histidines included in T1 Cu and T3 Cu were replaced by other amino acids. Histidine and cysteine bind to the T1 site, where substrate oxidation occurs, and coordinate its function [50]. These substitutions and deletions affect copper binding and electron transfer functions, which may result in *AaLac4*, *AaLac5*, and *AaLac7* showing differences in stability and activity compared to other enzymes within the family. Analysis of the SBL sequences of *AaLac* revealed that, in the B4-B5 region, serine (S) appeared in all sequences except *AaLac1, 2*, and *7*, where glycine (G) and alanine (A) were observed instead. In the B1–B2 region, *AaLac3* and *AaLac4* share the same sequence, which is also identical in *AaLac5* and *AaLac6*. This pattern is consistent with the phylogenetic tree results. Since the SBL can regulate laccase catalytic efficiency, it is suggested that *AaLac3**–**4* and *AaLac5**–**6* may have similar functions within their respective groups [51].

Understanding the tertiary structure of proteins provides crucial information for predicting their structural and functional roles, catalytic activities in chemical reactions, transportation, storage, and gene transcription regulation [52,53,54]. Predicting the precise binding affinity between small molecules and proteins is a significant challenge in drug development, and it can be achieved through deep learning based on protein–ligand binding affinity [55,56]. Consequently, protein tertiary structures can provide valuable insights into proteins for medical, pharmaceutical, and biotechnological industries [57,58,59]. However, previous studies on the laccase family in mushrooms have not included a detailed analysis of tertiary structures. In this study, we predicted the tertiary structure of the laccase family and identified the positions of histidine and cysteine residues. These residues are structurally and functionally significant in laccases due to their role in copper binding [1]. The histidine associated with T1 copper enables the oxidation of various substrates through non-covalent binding, and the His-Cys-His pathway facilitates electron transfer from T1 copper to the trinuclear cluster [60]. We identified the His-Cys-His motif within the tertiary structure of the laccase family. However, due to variations in the signature sequence L4, where deletions or substitutions occurred in histidine or cysteine residues, the motif was only partially observed in *AaLac5* and *AaLac7*. This serves as important evidence for explaining the functional differences arising from variations in the signature sequence. By analyzing the copper ion structure and enzymatic activity of the laccase family in future studies, we expect to gain a deeper understanding of how the positions of histidine and cysteine in the tertiary structure influence their structural and functional roles. Predicted tertiary structures of the *A. auricula-judae* laccase family and confirmed RSMD values revealed similar patterns among closely related laccases as observed in the phylogenetic tree. For example, *AaLac3* and *AaLac4*, which are shown to be closely related in the phylogenetic tree, exhibited low RMSD values, indicating similar tertiary structure patterns. This was also commonly observed in *AaLac5* and *AaLac6*. Laccases typically contain four copper ions and these copper ions play a crucial role in the stability of proteins [61,62]. Meanwhile, AlphaFold 2 generates the most likely structure based on sequence information if specific stoichiometry, ligands, or ions are not explicitly provided [28]. Thus, our predicted tertiary structure of the laccase family generated through AlphaFold 2 could not specify the location of copper ions, and we determined that additional experimental validation is necessary.

The tertiary structure can serve as foundational data for further studies on enzyme function by assessing binding affinity through molecular docking with substrates. The activity of laccase can be predicted through molecular docking with 2,2′-azino-bis-(3-ethylbenzothiazoline-6-sulfonic acid) (ABTS), a common substrate used to confirm laccase activity, and its potential to degrade toxic substances can be evaluated through docking with well-known toxic compounds such as Aflatoxin B1 (AFB1) [43,63,64]. Differences in substrate affinity can be utilized as a basis for functional classification within the laccase family. Additionally, molecular docking can be employed to analyze the molecular reaction mechanisms between enzymes and substrates [65]. Therefore, if laccase tertiary structure data are obtained, it is possible to analyze the function of laccase through in silico work without performing actual experiments. Our method can quickly and easily identify laccase in organisms with unknown genetic information or those that have undergone random mutations. This can be used as valuable data for understanding the structure and function of laccase.

## 4. Materials and Methods

### 4.1. Strain Cultivation and RNA Extraction

In this experiment, the *Auricularia auricula* variety Yong-A developed at the Jeollanam-do Agricultural Research & Extension Service was used. *A. auricula-judae* was cultured on PDA for 14 days at 25 °C and then inoculated onto a sawdust medium composed of 80% sawdust and 20% rice bran (*w*/*w*). The inoculated medium was incubated at 25 °C for 30 days to allow for mycelial growth, followed by fruiting body formation under conditions of 18 °C temperature, 90% relative humidity, and 2000 ppm CO_2_. Mature fruiting bodies were harvested, immediately frozen in liquid nitrogen, and stored at −80 °C. RNA extraction was performed following a standardized protocol using the RNA purification kit (GeneAll Biotechnology, Ribospin II, Seoul, Republic of Korea).

### 4.2. RNA Sequencing, Assembly, and Annotation

RNA sequencing of *A. auricula-judae* was performed by Theragenbio (Seongnam, Republic of Korea) using the NovaSeq6000 platform (Illumina, Inc., San Diego, CA, USA). Following sequence decoding, assembly was conducted using the Trinity v2.15.1 software [66]. Subsequently, the TransDecoder v5.7.1 program [67] was employed to predict coding sequences (CDS).

### 4.3. Identification of Laccase Genes in A. auricula-judae

Genes belonging to the laccase family are known to contain Cu-Oxidase, Cu-Oxidase_2, and Cu-Oxidase_3 (PF00394, PF07731, PF07732) domains. Hidden Markov model (HMM) profiles were downloaded from InterPro (https://www.ebi.ac.uk/interpro/download/Pfam/, accessed on 16 January 2024), and CDS generated through RNA sequencing were searched using HAMMER software version 1.3 [68] with an expected value (E-value) < 1 × 10^−5^. As a result, 19 putative laccase genes with all three domains were discovered. To reduce redundancy, sequences with over 90% identity were removed using Decrease Redundancy (https://web.expasy.org/decrease_redundancy/, accessed on 16 January 2024), resulting in 7 defined protein sequences from the initial 19 putative laccase genes. The 7 genes, each containing all three domains, were classified as members of the Laccase family and named *AaLac1* through *AaLac7*. These 7 laccase genes obtained in this study have been submitted to NCBI’s GenBank (PP337285 to PP337291).

### 4.4. Analysis of A. auricula-judae Laccase Protein Sequence

N-glycosylation sites were identified using NetNGlyc-1.0 (https://services.healthtech.dtu.dk/service.php?NetNGlyc-1.0, accessed on 16 January 2024). Protein secondary structures of the Laccase family genes were predicted using SOPMA in NPS@ (https://npsa.lyon.inserm.fr/cgi-bin/npsa_automat.pl?page=/NPSA/npsa_sopma.html, accessed on 16 January 2024). Amino acid sequences of laccase from other organisms were retrieved from the RCSB PDB (Research Collaboratory for Structural Bioinformatics Protein Data Bank).

### 4.5. Alignment of Protein Sequences and Phylogenetic Analysis

To visualize the protein sequences of the laccase family genes, protein sequences were aligned using MAFFT 7 (https://mafft.cbrc.jp/alignment/server/index.html, accessed on 16 January 2024), and subsequently visualized using GeneDoc 2.7 [69]. Phylogenetic trees were constructed using the Neighbor-Joining (NJ) method in MEGA 11, applying the Poisson model for amino acid substitution. Branch robustness was assessed through bootstrap analysis with 1000 replicates. Laccase sequences from other organisms were retrieved from the NCBI (National Center for Biotechnology Information) database and included in the phylogenetic analysis.

### 4.6. Tertiary Structure of Laccase Proteins

Google DeepMind’s AlphaFold 2 was used to predict the tertiary structure of *AaLac* [28]. The predicted protein’s tertiary structure was visualized using PyMOL v2.5, and the RMSD of the laccase family structures was calculated under cycle 5 and cutoff 2.0 Å conditions [70]. For protein tertiary structure validation, PROCHECK (https://saves.mbi.ucla.edu/, accesed on 1 November 2024), ProSA-web (https://prosa.services.came.sbg.ac.at/prosa.php, accesed on 23 October 2024), and QMEAN (https://swissmodel.expasy.org/qmean/, accesed on 23 October 2024) were used.

## 5. Conclusions

In this study, we identified members of the laccase family in *A. auricula-judae* and analyzed their characteristics and structures based on sequence data. We predicted potential functional differences among laccases within the family based on N-glycosylation sites. Subsequently, by predicting the protein tertiary structure, we established a foundation for predicting the function of *AaLac*. Our method enables the easy and rapid identification and analysis of laccases in many organisms with unknown genetic information through RNA sequencing, allowing for the analysis of the functional characteristics of the enzyme. This approach is expected to enable the efficient and cost-effective discovery of new enzymes and the exploration of their functions.

## Figures and Tables

**Table 1:** Characteristics of the *A. auricula-judae* laccase family.

Laccase	Coding Sequence Size (bp)	Complete CDS	Protein Length (aa)	Molecular Weight (KDa)	Number of Potential N-Glycosylation Sites
*AaLac1*	1779	Y	532	58.13	2
*AaLac2*	1896	Y	571	63.77	4
*AaLac3*	1755	Y	524	58.19	8
*AaLac4*	1470	Y	429	47.79	5
*AaLac5*	1545	Y	455	49.37	4
*AaLac6*	1731	Y	516	56.79	4
*AaLac7*	1680	Y	499	54.46	10
1GW0	-	-	559	61.79	9
1GYC	-	-	499	53.64	5
1KYA	-	-	499	53.34	4
2QT6	-	-	498	53.95	5

1GW0; *Melanocarpus albomyces,* 1GYC; *Trametes versicolor* laccase I, 1KYA; *T. versicolor* laccase III, 2QT6; *Lentinus tigrinus*.

**Table 2:** The predicted secondary structures of *A. auricula-judae* laccase.

Laccase	α-Helix (%)	Extended Strand (%)	β-Turn (%)	Random Coils (%)
*AaLac1*	7.33	25.94	5.26	61.47
*AaLac2*	7.01	28.37	5.60	59.02
*AaLac3*	6.87	28.63	5.34	59.16
*AaLac4*	8.16	27.74	4.90	59.21
*AaLac5*	3.52	30.77	5.71	60.00
*AaLac6*	6.01	29.26	6.01	58.72
*AaLac7*	4.21	30.66	5.01	60.12
1GW0	5.72	26.12	6.08	62.08
1GYC	5.61	29.26	6.41	58.72
1KYA	5.61	29.66	5.01	59.72
2QT6	5.82	30.52	6.22	57.43

1GW0; *Melanocarpus albomyces*, 1GYC; *Trametes versicolor* laccase I, 1KYA; *T. versicolor* laccase III, 2QT6; *Lentinus tigrinus*.

**Table 3:** Quality estimations of laccase family tertiary structure.

Laccase Family	Oligomeric State	Ramachandran plot ^a^ (%)	ProSA Z-Score	QMEAN Score
*AaLac1*	Monomer	84.9	−7.94	0.56
*AaLac2*	Monomer	80.8	−7.16	0.69
*AaLac3*	Monomer	89.0	−7.5	0.75
*AaLac4*	Monomer	90.2	−6.71	0.71
*AaLac5*	Monomer	88.5	−7.8	0.76
*AaLac6*	Monomer	89.2	−8.1	0.77
*AaLac7*	Monomer	88.1	−8.55	0.67

^a^ Results are given on the basis of most favored regions.

**Table 4:** Structure comparison of laccase family tertiary structures through RMSD (Root mean square deviation).

Å	*AaLac1*	*AaLac2*	*AaLac3*	*AaLac4*	*AaLac5*	*AaLac6*	*AaLac7*
*AaLac1*		2.356	1.600	2.830	1.131	0.976	1.203
*AaLac2*	2.239		0.407	0.488	0.449	0.456	0.893
*AaLac3*	1.925	0.407		0.182	0.430	0.404	0.956
*AaLac4*	4.222	0.488	0.182		0.519	0.463	1.046
*AaLac5*	1.131	0.449	0.430	0.519		0.228	0.873
*AaLac6*	0.976	0.456	0.404	0.463	0.228		0.847
*AaLac7*	1.201	0.893	0.956	1.046	0.873	0.847	

## Data Availability

The data are available from the corresponding author upon reasonable request.
